# Advances in thiosemicarbazone metal complexes as anti-lung cancer agents

**DOI:** 10.3389/fphar.2022.1018951

**Published:** 2022-09-27

**Authors:** Xian-Guang Bai, Yunyun Zheng, Jinxu Qi

**Affiliations:** School of Medicine, Pingdingshan University, Pingdingshan, China

**Keywords:** thiosemicarbazone, schiff base, metal complexes, anti-lung cancer, chemotherapy

## Abstract

The great success of cisplatin as a chemotherapeutic agent considerably increased research efforts in inorganic biochemistry to identify more metallic drugs having the potential of treating lung cancer. Metal coordination centres, which exhibit a wide range of coordination numbers and geometries, various oxidised and reduced states and the inherent ligand properties offer pharmaceutical chemists a plethora of drug structures. Owing to the presence of C=N and C=S bonds in a thiosemicarbazone Schiff base, N and S atoms in its hybrid orbital has lone pair of electrons, which can generate metal complexes with different stabilities with most metal elements under certain conditions. Such ligands and complexes play key roles in the treatment of anti-lung cancer. Research regarding metallic anti-lung cancer has advanced considerably, but there remain several challenges. In this review, we discuss the potential of thiosemicarbazone Schiff base complexes as anti-lung cancer drugs, their anti-cancer activities and the most likely action mechanisms involving the recent families of copper, nickel, platinum, ruthenium and other complexes.

## Introduction

Lung cancer is a malignant tumour that develops from the bronchial mucosa or glands of the lung, which poses the greatest threat to human health and lives, exhibiting the fastest increase rate in terms of morbidity and mortality (Brody, 2014). In 2020, ∼19.3 million new cancer cases and approximately 10 million cancer-related deaths were reported worldwide (Sung et al., 2021). Lung cancer remains the leading cause of cancer-related death, engendering an estimated 1.8 million deaths (18%) with an estimated 2.2 million new cases (11.4%) in 2020 (Sung et al., 2021). The dramatic increase in the cases of lung cancer and multidrug-resistant infections has necessitated the search for novel treatment options and strategies (Tsai et al., 2018). New small-molecule anti-cancer agents Exhibit great potentials. However, the frequent occurrence of multidrug resistance (MDR) in lung cancer warrants the development of specific agents that can overcome MDR (Barrera-Rodriguez and Fuentes, 2015). Metal-based drugs are structurally stable and have unique three-dimensional configurations, which can be effectively used to treat multidrug-resistant infections (Rostan et al., 2021).

Thiosemicarbazone Schiff bases have garnered a lot of interest due to their multifunctional metal-chelating properties, inherent biological activities and structural flexibility (Matesanz et al., 2021a). It was established that thiosemicarbazides were metal chelators before the discovery of their anti-tumour activity. In addition to the presence of the C=N and C=S bonds in their own structure, which is favourable for metal coordination, it possesses a flexible thiourea structure that can introduce different substituents or functional groups (Scarim and Chin, 2022a). Especially for various heterocyclic ligands, introducing more heteroatoms enriches thiosemicarbazone’s coordination mode and enhances its coordination ability (Stefani et al., 2013). They utilize the N–N–S donor system of ligands to form stable complexes with transition metals. Many studies reported that these complexes exhibited higher anti-tumour activity than ligands *in vitro* and *in vivo* and were extensively employed in the field of anti-tumour research (Summers, 2019a). Metal complexes are biologically and chemically diverse, unlike their corresponding ligands. This diversity is not only reflected in the metal and its oxidation state but also in the diversity of ligands that form coordination bonds with it and the different coordination modes (Matesanz et al., 2021a). Many literatures report that thiosemicarbazone ligands and their metal complexes have significant antitumor activity against lung cancer, liver cancer, colon cancer, breast cancer, neuroma and other tumors (Onodera et al., 2007; Lovejoy et al., 2012; Maqbool et al., 2020; Dharmasivam et al., 2022). Among many cancers, lung cancer has been the most common disease affecting human health. Thiosemicarbazone ligands have been reported to exhibit significant antitumor activity against lung tumor xenografts and have no significant toxicological effects, both intravenously and orally routes (Lovejoy et al., 2012). Therefore, it is necessary to review the research progress of thiosemicarbazone metal complexes in the treatment of lung cancer. We hope that this review guides researchers to better understand the application of metal-based agents in anti-lung cancer treatment and provide new ideas for the design and research of anti-tumour agents.

## Thiosemicarbazone ligands in a clinical trial

**SCHEME 1:**
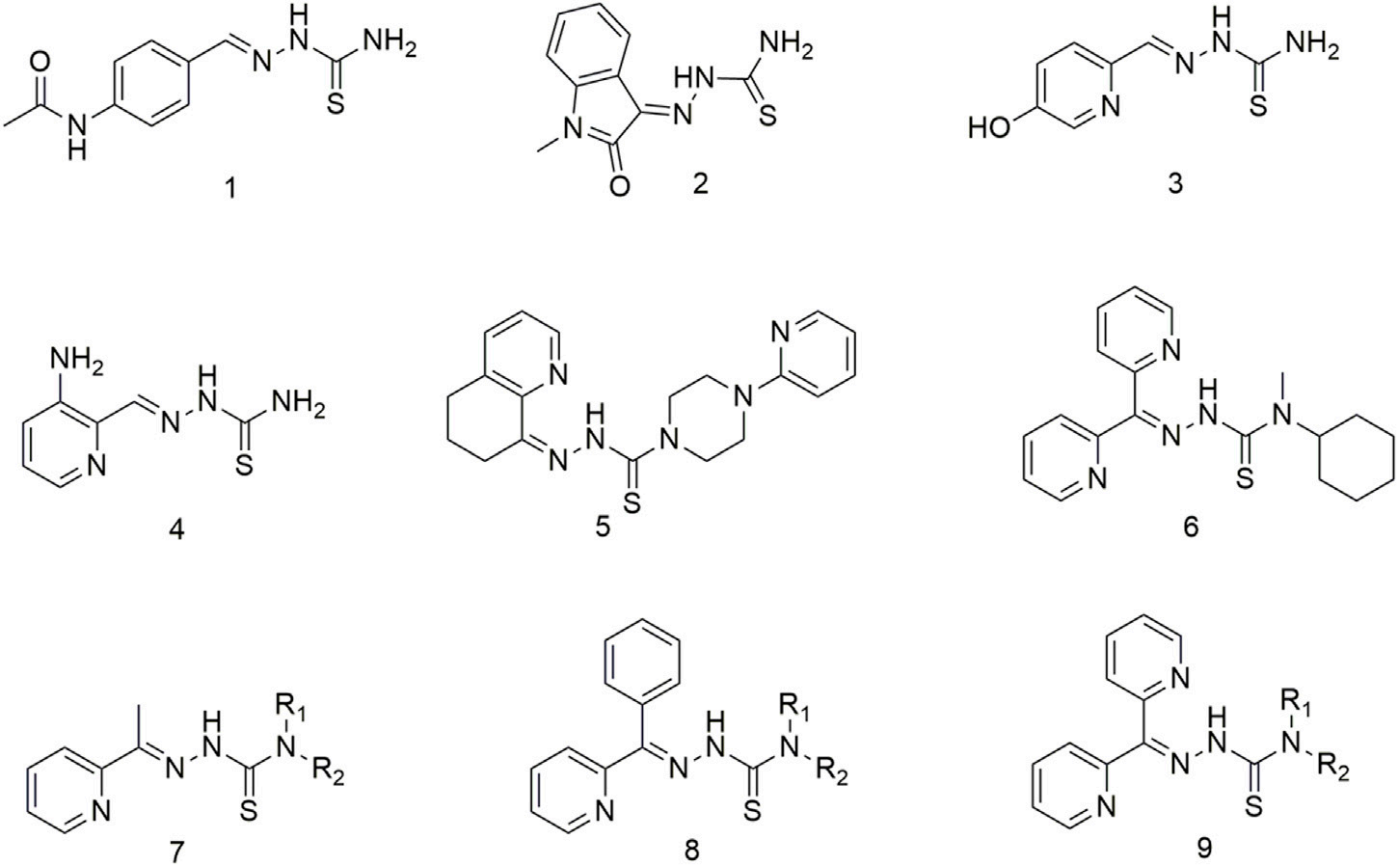
Thiosemicarbazone ligands for clinical application.

The thiosemicarbazone compounds used in clinical research include acetamidobenzaldehyde semicarbazide (**1**), N-methylisatin thiosemicarbazone (**2**), 5-hydroxy-2-methylcarbamate acylpyridine thiosemicarbazide (**3**), 3-aminopyridine-2-carbaldehyde thiosemicarbazide (**4**), COTI-2 (**5**), bis-2-pyridyl ketone 4-cyclohexyl-4-methyl-3-thiosemicarbazide (**6**) (Scheme 1) (Summers, 2019a; Matesanz et al., 2021a; Scarim and Chin, 2022a). Triapine is the most prevalent anti-cancer agent and has been included in over 20 clinical trials for treating multiple cancers, including pancreatic cancer, non-small cell lung cancer (NSCLC), leukaemia and myeloproliferative diseases (Scarim and Chin, 2022a). However, some tumour types respond poorly to Triapine, developing serious side effects, such as hyperhemoglobinemia (Yu et al., 2006; Yu et al., 2009). Among this class of compounds, the 2-acetylpyridine thiosemicarbazone series (**7**) demonstrated significant anti-cancer activity (Onodera et al., 2007). Initial studies have shown that thiosemicarbazone can inhibit ribonucleotide reductase activity and further impede DNA synthesis, finally exerting its anti-cancer effect (Kowol et al., 2009; Yu et al., 2011). Further studies have shown that the anti-tumour activity of thiosemicarbazone is due to the joint action of multiple mechanisms (de Siqueira et al., 2019a). Thiosemicarbazone ligands have been widely reported to inhibit cellular iron uptake and affect cellular iron metabolism, and up-regulation of the downstream regulatory gene 1 of the metastasis suppressor protein N-myc (Serda et al., 2014; Malarz et al., 2018). Moreover, compound **9** causes DNA damage and inhibition of selective cellular topoisomerase IIɑ (Rao et al., 2009). This kind of a ligand is a good metal chelator, and the complexes formed by metal coordination exhibit redox activity and can catalyse hydrogen peroxide formation *in vivo* to generate reactive oxygen species (ROS) (Mckenzie-Nickson et al., 2016; Matesanz et al., 2021a). Through extensive structure-activity relationship studies, a new series of 2-benzoylpyridine thiosemicarbazone (**8**) incorporated 2-benzoylpyridine moieties in the structural backbone of previous arylhydrazone (Stefani et al., 2013). These compounds exhibited potent anti-lung cancer and anti-metastatic activities (Wu et al., 2006; Mahale et al., 2015). A series of di-2-pyridyl ketone thiosemicarbazone (**9**) were developed (Lovejoy et al., 2012) to improve the efficacy and safety of these potential anti-tumour drugs. This new compound exhibits markedly selective activity *in vivo* against human lung cancer (DMS-53) xenografts *via* the intravenous and oral routes (Lovejoy et al., 2012). Importantly, these analogues did not cause cardiotoxicity in tumour-bearing nude mice at high doses (Wangpu et al., 2016). To overcome MDR, di-2-pyridyl ketone thiosemicarbazone analogues target lysosomes by ‘hijacking’ the MDR pump p-glycoprotein (Pgp), which has potent anti-tumour and anti-metastatic activities both *in vitro* and *in vivo* (Wangpu et al., 2016). However, high doses of di-2-pyridyl ketone thiosemicarbazone may cause cardiac fibrosis, which warrants the design and synthesis of new thiosemicarbazone compounds. In second-generation thiosemicarbazone compounds wherein the terminal H of N4 was substituted with an alkyl group, compound 6 showed potential application prospects in antitumor (Lovejoy et al., 2012). Compound **6** exhibits higher anti-tumour activity *in vivo* and demonstrates considerable improvement in tolerability when administered orally or intravenously compared with first-generation thiosemicarbazone compounds (Stariat et al., 2013; Maqbool et al., 2020; Dharmasivam et al., 2022). Thiosemicarbazones combine with intracellular copper (Cu) ions to produce stable Cu complexes once they are inside the lysosome. These complexes generate ROS through the redox cycle, causing the lysosomal membrane to collapse (LMP), ultimately inducing apoptosis (Zhao et al., 2017; Gu et al., 2019).

## Thiosemicarbazone copper complexes

**SCHEME 2:**
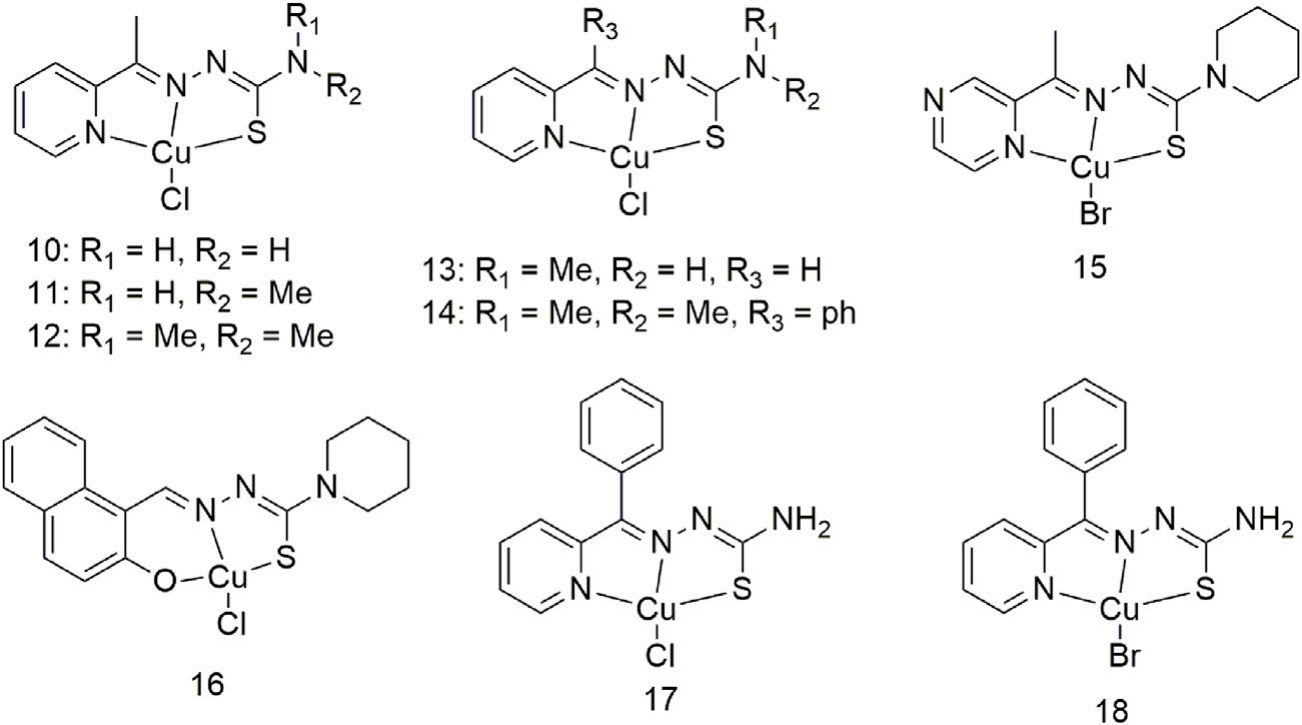
Structures of the copper (II) complexes (**10–18**) of thiosemicarbazone.

Three Cu (II) complexes (**10–12**, Scheme 2) were synthesised and characterised to show that thiosemicarbazone Cu (II) complexes demonstrate strong anti-tumour activity (Qi et al., 2020). The anti-proliferative activity of complex 12 on the human lung cancer cell line A549 was the highest (0.20 ± 0.04 mM) of all the complexes. The lipophilicity of thiosemicarbazone ligands is closely related to the anti-proliferative activity of Cu (II) complexes on lung cancer cells Studies regarding the cellular mechanism of such Cu (II) complexes have shown that they promote apoptosis by catalysing hydrogen peroxide to generate intracellular ROS. Researchers proposed to develop two Cu (II) complexes based on the His242 residue of the IIA subdomain of the human serum albumin (HSA) carrier (**13** and **14**, Scheme 2) (Qi et al., 2016) to improve the delivery efficiency, anti-cancer activity and selectivity of anti-cancer metal preparations *in vivo*. Complex 13 binds to HSA subdomain IIA by hydrophobic interaction, while complex **14** binds to HSA by coordinating with His242. The cytotoxicity of complex 14 toward the A549 cell line (0.15 ± 0.01 μM) was significantly higher than that of cisplatin (17.36 ± 0.25 μM), and it significantly inhibited the growth of A549 tumours in nude mice. The anti-tumour activity against human large cell lung cancer cells (NCI-H460) of complexes **15** and **16** increased more than 40-fold compared with the ligands and exhibited significant pro-apoptotic activity at nanomolar concentrations (Scheme 2). Complexes **15** and **16** mediate significant antitumor activity by generating reactive oxygen species (ROS) through the redox cycle. (Scheme 2). Excess ROS can lead to the dissipation of mitochondrial membrane potential and promote the release of mitochondrial apoptotic factors. Complexes **17** and **18** also show effective oxidative cleavage of supercoiled DNA in the presence of hydrogen peroxide and exhibit good anti-tumour activity against the NCI-H460 cell line with IC_50_ values in the range of 0.08–1.98 μM, which are higher than cis-Platinum (Pt) is 83 times lower (Scheme 2) (Liu et al., 2016).

**SCHEME 3:**
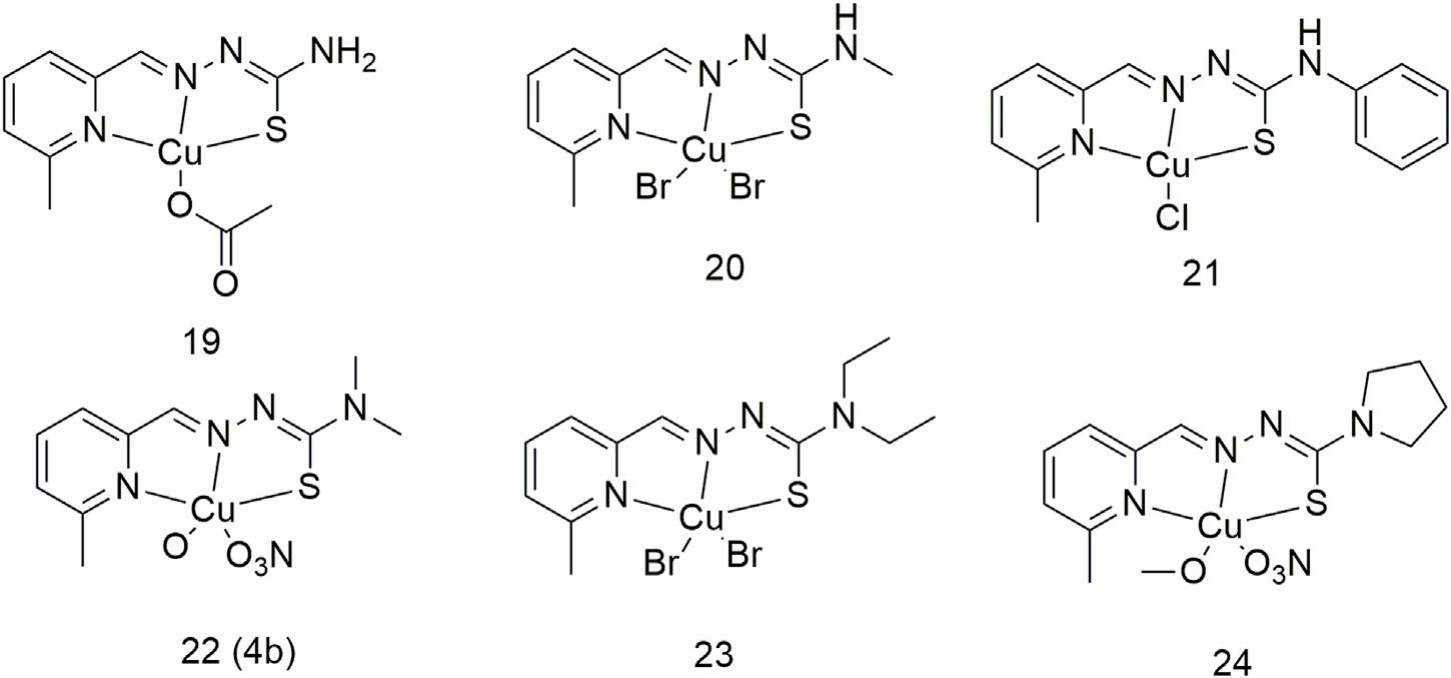
Structures of the copper (II) complexes (**19–24**) of thiosemicarbazone.

By assessing the structure–activity relationship of six Cu (II) complexes (**19–24**, Scheme 3), researchers confirmed the multi-target ability of complex **22** on proteins and DNA (Wang et al., 2018). These six Cu complexes showed higher cytotoxicity against lung cancer cells (A549 and NCI-H460) than the ligands. The toxicity of complexes **19–24** toward the A549 cell line (≤13.69 ± 0.97 μM) was significantly higher than that of cisplatin (17.36 ± 0.75 μM). Importantly, in the A549 cell line, the cytotoxicity of the Cu (II) complex was enhanced by 1.0–3.0-fold upon binding to HSA. These six complexes inhibit cancer cell growth mainly by targeting DNA and apoptosis-related proteins in tumours. Forming a complex with HSA can improve the delivery efficiency of Cu complexes and impart a stronger ability to inhibit tumour growth.

**SCHEME 4:**
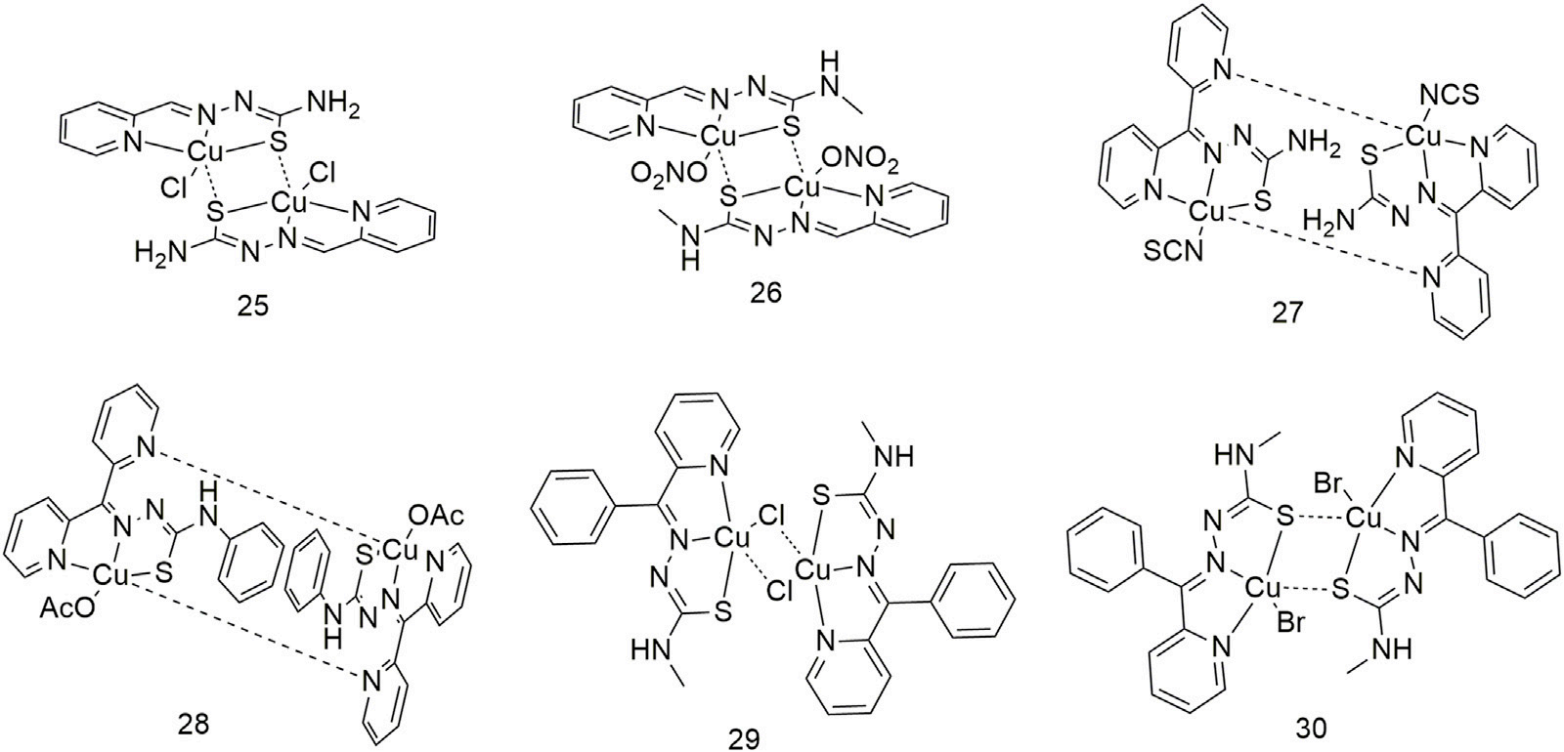
Structures of copper (II) complexes (**25–30**) of thiosemicarbazone.

It is essential to conduct research regarding Cu complexes to produce a new generation of metallic anti-cancer drugs. Therefore, four dinuclear Cu (II) complexes (**25–28**, Scheme 4) were synthesised using Schiff base thiosemicarbazides (Qi et al., 2015). The anti-proliferative activity of these four Cu complexes on lung cancer cells (A549 and NCI-H460) was stronger than that of the ligands. Among them, the cytotoxic activity of complex **28** was the highest, with IC_50_ values of 0.507 ± 0.021 μM and 0.235 ± 0.010 μM for the A549 and NCI-H460 cell lines, respectively. Binuclear structures and their cytotoxicity are also shown in complexes **29** and **30** (Scheme 4); IC_50_ values are at the nanomolar level and 83 times lower than cisplatin. Their cytotoxicity is significantly more enhanced than that of the corresponding ligands (Liu et al., 2016).

**SCHEME 5:**
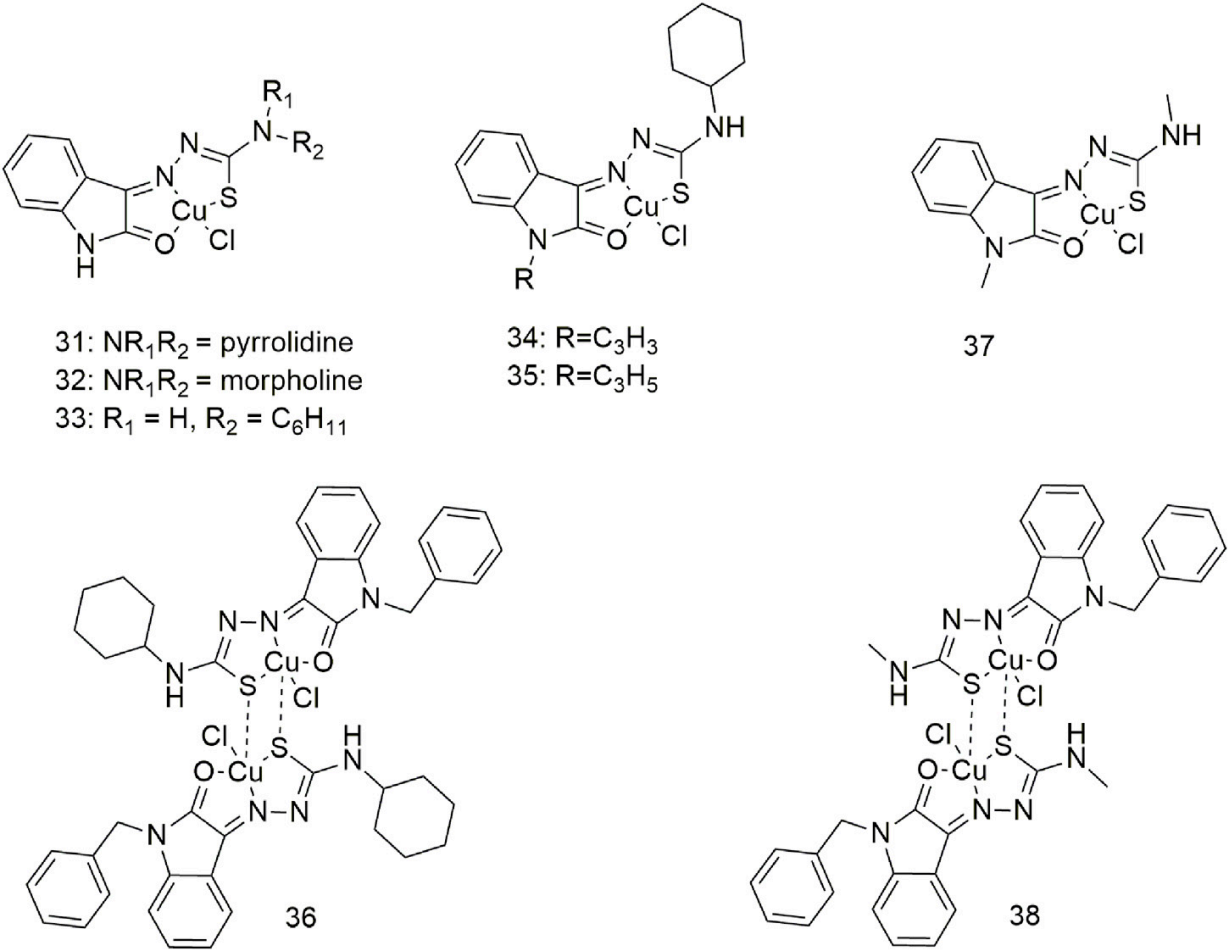
Structures of the copper (II) complexes (**31–38**) of thiosemicarbazone.

Three complexes (**31–33**, Scheme 5) with broad-spectrum anti-tumour activity are formed when Cu (II) coordinates with 5-methoxyisatin thiosemicarbazone ligands with different N-terminal substituents; the IC_50_ value of complex **31** for the A549 cell line is 17.88 ± 0.16 μM (Aneesrahman et al., 2019). Complexes **34–36** act as an intercalating agent toward DNA and as a binding agent toward bovine serum albumin (BSA), **(** Scheme 5) (Mathiyan et al., 2016). The *in vitro* cytotoxicity study of complex **36** showed good anti-proliferative activity against the A549 cell line (Mathiyan et al., 2016). Molecular docking studies have shown that complexes **37** and **38** formed hydrogen bonds and hydrophobic interactions with the tyrosinase kinase receptor (Scheme 5) (Haribabu et al., 2021). These complexes can generate morphological changes in the A549 cell line based on fluorescence microscopy and show cytotoxic activity against the A549 cell line with IC_50_ values of 59.50 and 29.51 µM, respectively.

## Thiosemicarbazone ruthenium complexes

**SCHEME 6:**
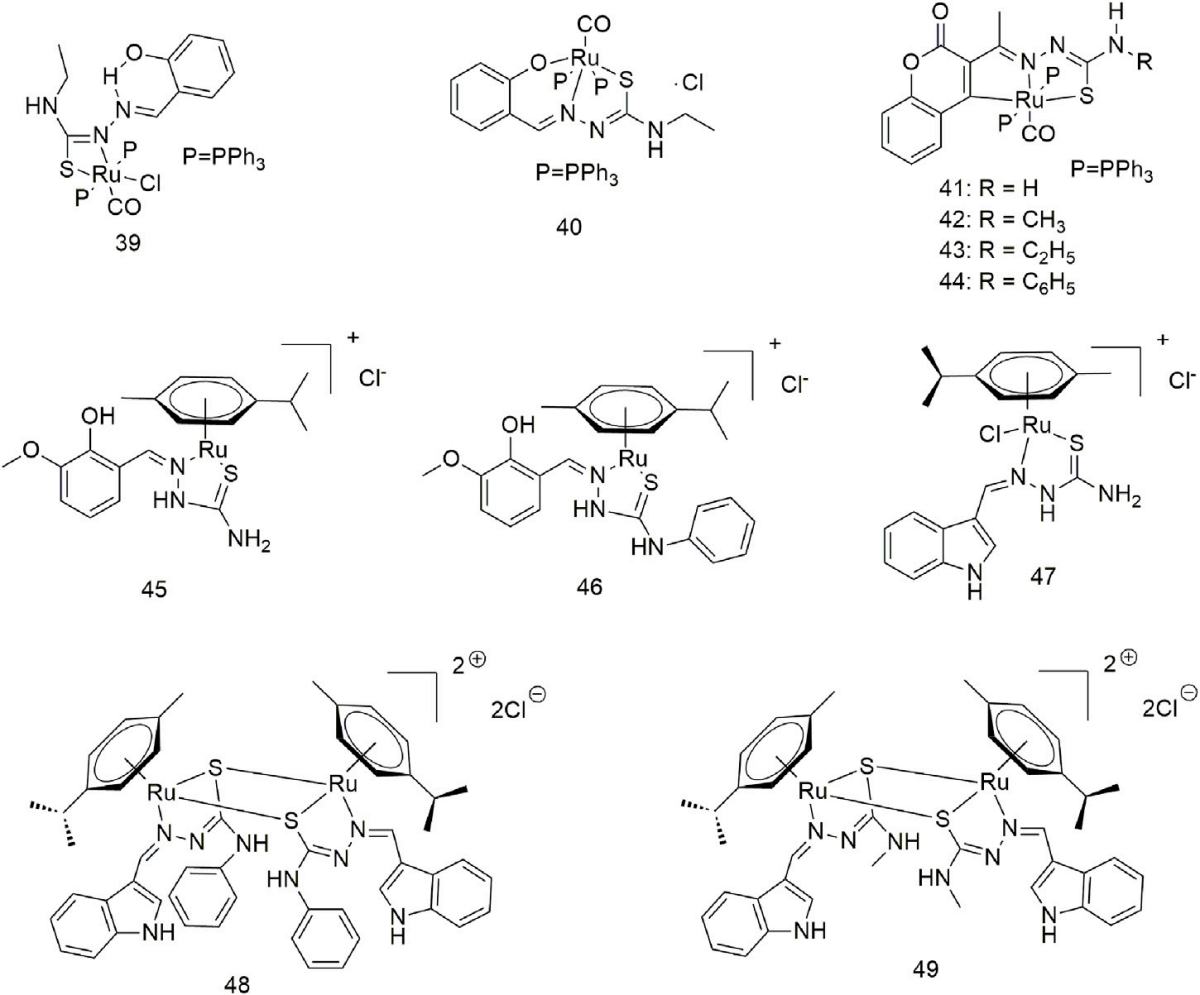
Structures of ruthenium complexes (**39–49**) of thiosemicarbazone.

Salicylaldehyde-4-ethylthiosemicarbazide was reacted with [RuHCl(CO) (PPh_3_)_3_] to produce complexes **39** and **40** (Scheme 6) (Kalaivani et al., 2014). Complexes **39** (20 ± 1.10 μM) and **40** (17 ± 0.93 μM) showed higher IC_50_ values for the A549 cell line compared with standard cisplatin (25 ± 1.11 μM). Complexes **39** and **40** are absorbed intracellularly and bind to the protein lysozyme, releasing CO and generating cytotoxicity. Four new divalent organoRu complexes (**41–44**, Scheme 6) were synthesised by modifying different groups at the N4 position of 3-acetylcoumarin-4-substituted thiosemicarbazide (Kalaiarasi et al., 2018). These complexes are more active than cisplatin and nontoxic to the human normal keratinocyte cell line HaCaT. The activity of complex **43** was optimal due to the N-terminal modification of thiourea with a more electron-donating ethyl group. Anna Gatti et al. reported the structures of two semi-sandwich aromatic Ru (II) thiosemicarbazide complexes (**45** and **46**, Scheme 6) with remarkable anti-tumour proliferative activity (Gatti et al., 2018). It forms mononuclear (**47**) and dinuclear complexes (**48** and **49**) with [RuCl_2_(p-cymene)]_2_ in different reactions using indole thiosemicarbazide as a ligand, which is water soluble **(** Scheme 6) (Haribabu et al., 2018; Haribabu et al., 2021). These complexes exhibit significant cytotoxic activity against the A549 cell line *via* apoptosis, but not normal cells (L929). Most likely due to its higher DNA/protein binding affinity, binuclear Ru (η^6^-p-cymene) complex shows better activity than Mono-Ru (η^6^-p-cymene) complex. Complex 49 (IC_50_ = 11.5 μM) was significantly more active on the A549 cell line than homogeneous cisplatin (IC_50_ = 21.3 μM).

**SCHEME 7:**
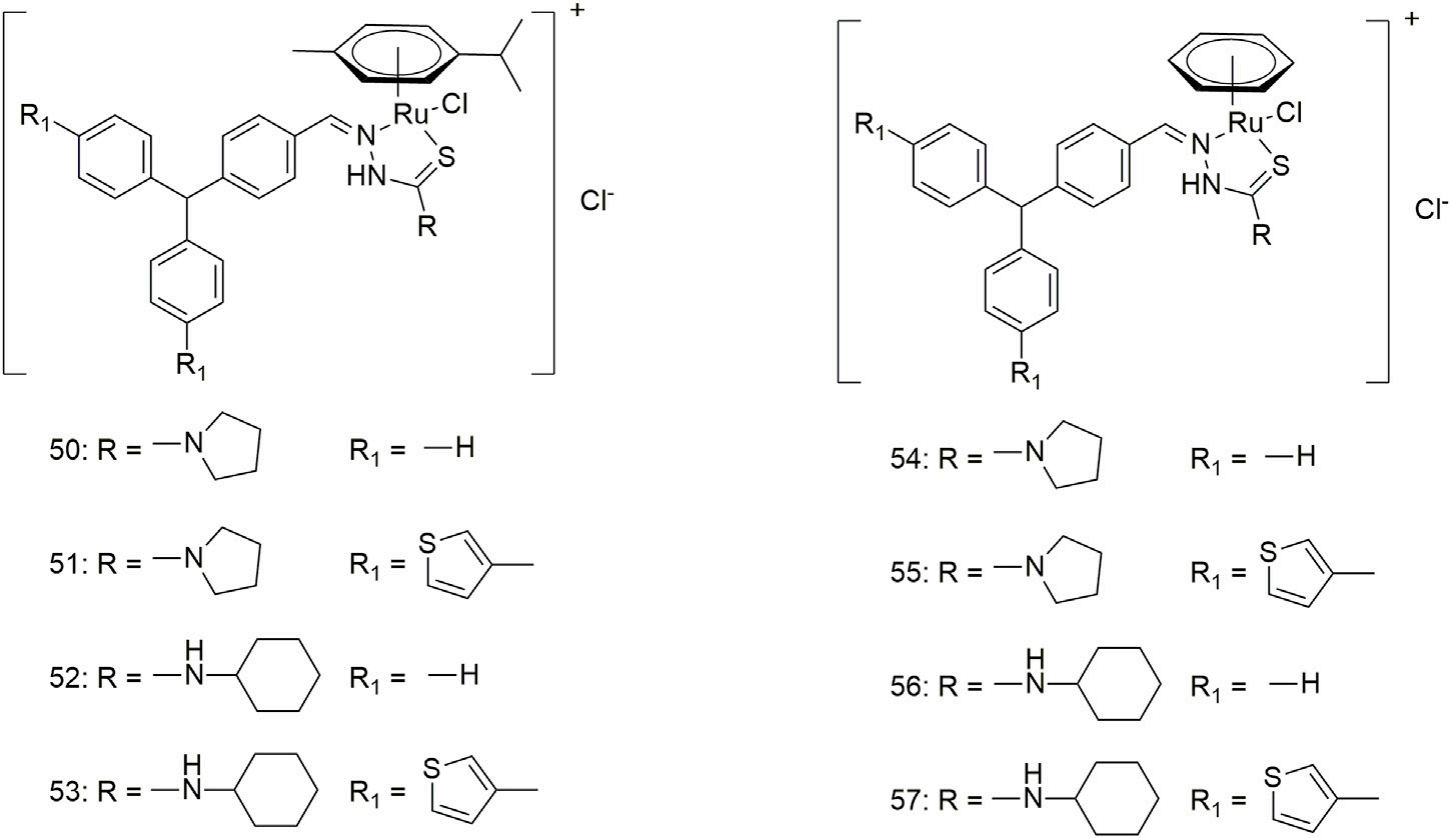
Structures of ruthenium complexes (**50–57**) of thiosemicarbazone.

Eight triarylamine-thiosemicarbazides Ru (II)-arene semi-sandwich complexes (**50–57**, Scheme 7) have been reported to bind DNA/HSA, generating significant cytotoxic activity (Muralisankar et al., 2019). The anti-tumour activity of these Ru (II) complexes on the A549 cell line was significantly different. The IC_50_ values of complexes **50**, **52** and **57** were greater than 100 μM, while complex **54** showed optimal anti-tumour activity (7.24 ± 5.4 μM). Furthermore, the treatment of the A549 cell line with complex **54** (10 μM) for 24 h produced significant changes in cell morphology, nuclear condensation and cell shrinkage, suggesting that cell death occurs *via* apoptosis under the action of this complex.

## Thiosemicarbazone nickel (II) complexes

**SCHEME 8:**
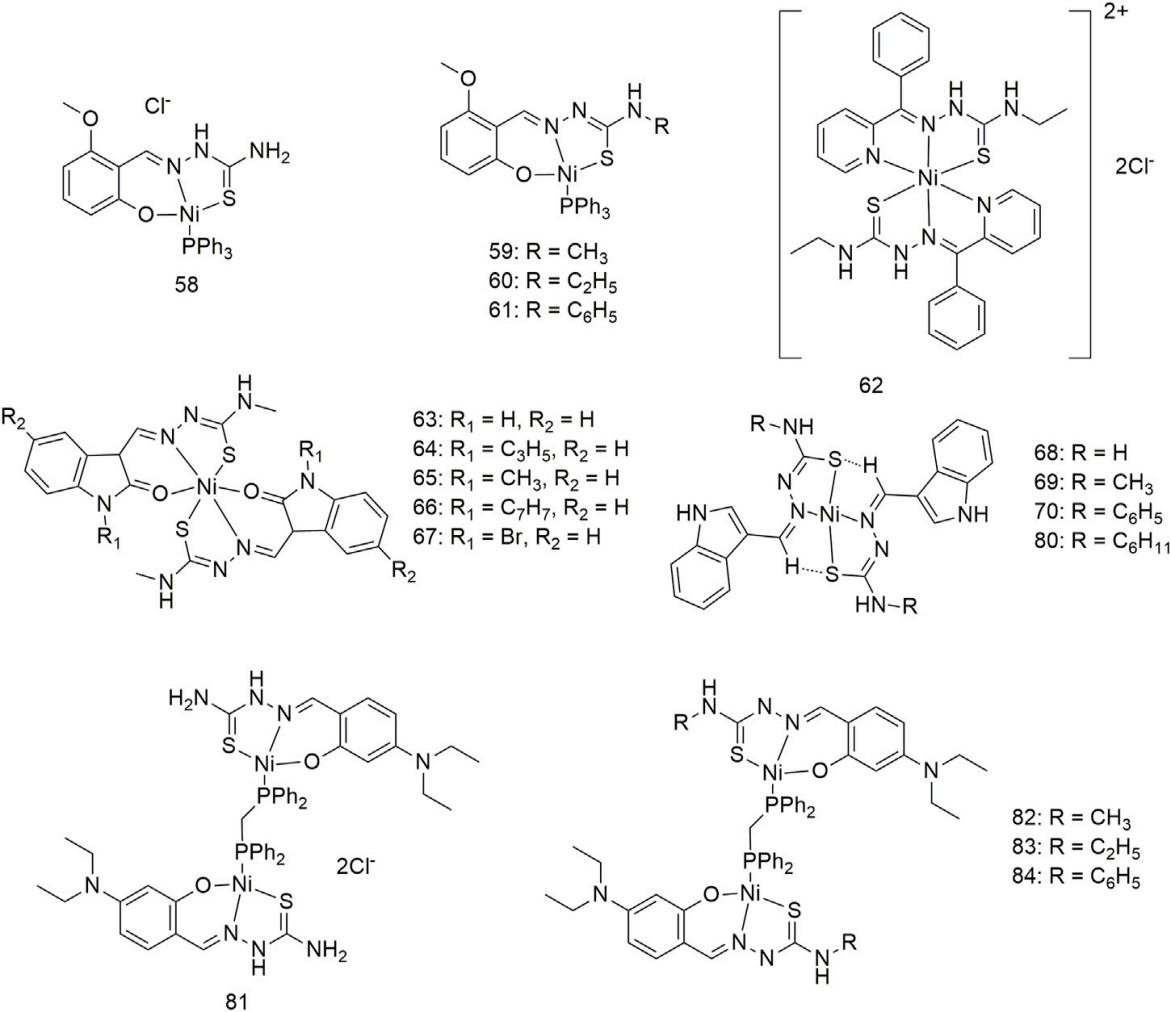
Structures of nickel (II) complexes (**58–84**) of thiosemicarbazone.

Complexes **58–61** bind to calf thymus (CT)-DNA in an intercalated manner and interact with BSA in a statically quenched manner (Scheme 8) (Kalaivani et al., 2014a). Complexes **58–61** show significant anti-proliferative activity (IC_50_, 27–30 μM) in the case of the A549 cell line under certain experimental conditions. The anti-tumour mechanisms of these complexes may be ROS-super-generation and lipid peroxidation, resulting in decreased mitochondrial membrane potential, caspase-3 activation and DNA fragmentation. Therefore, complexes **58–61** can cause apoptosis in the A549 cell line in a mitochondria-mediated manner and inhibit the migration and metastasis of lung cancer cells. Additionally, complex **62** interacts with CT-DNA and does not require any external force to cleave the DNA (Scheme 8) (Mathiyan et al., 2016). Complex 62 can bind to BSA and change the secondary structure of the protein. Complex **62** exhibits significant activity against the A549 cell line with an IC_50_ value of 80.10 μM and is less toxic to the L929 cell line. Complexes **63–67** can interact with CT-DNA and BSA, cleave DNA without external factors and change the secondary structure of proteins (Scheme 8) (Haribabu et al., 2015). These five complexes have significant activity (IC_50_ < 30 μM) against the A549 cell line, with complex 64 showing an IC_50_ value of less than 0.1 μM. Complexes **68–80** show high antioxidant activity and antihemolytic activity against 2-2-diphenyl-1-picrylhydrazyl (DPPH) free radicals (Haribabu et al., 2017). The four complexes shows excellent anti-tumour activity (Scheme 8) (IC_50_, 45.1–57.2 μM) against lung cancer (A549) and low toxicity (IC_50_ > 600 μM) against mouse embryonic fibroblasts (L929). Intercalation interactions of four binuclear Ni (II) complexes (**81–84**) with DNA were confirmed by ethylene bromide shift studies and DNA viscosity measurements (Scheme 8) (Kalaiarasi et al., 2020). The interaction mechanism of complexes **81–84** with BSA is static. The four binuclear Ni (II) complexes exhibited significant cytotoxicity against the A549 cell line with lower IC_50_ values (4.97–6.44 μM) than the standard metal drug cisplatin (IC_50_, 31.08 ± 0.79 μM).

## Thiosemicarbazone platinum (II), gold (I) and silver (I) complexes

**SCHEME 9:**
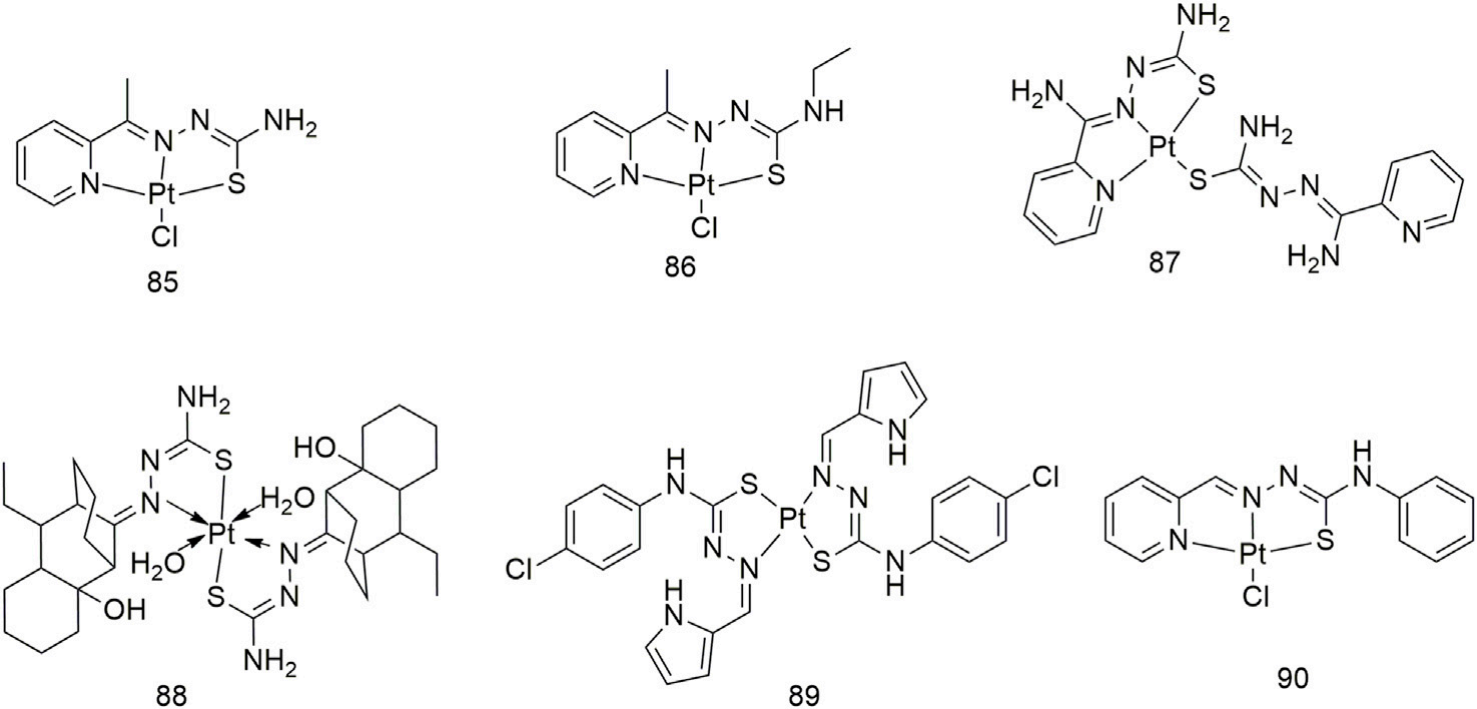
Structures of platinum (II) complexes (**85–90**) of thiosemicarbazone.

Cisplatin has become a widely used anti-cancer drug in clinics. Current research shows the action mechanism of involves the hydrolysis of cisplatin in the human body, removal of two chlorine atoms and binding to two purines on the genomic DNA and twisting the DNA double helix structure, thereby inhibiting DNA replication and transcription and ultimately causing cancer cell apoptosis (Pahontu et al., 2016a). The quenching mechanism of HSA by three thiosemicarbazide Pt (II) complexes (**85–87**) may be a static binding mode and impact the microenvironment of tryptophan residues in HSA (Scheme 9) (Lin et al., 2017). These three complexes show significant anti-proliferative activity (IC_50_, 2.8–9.6 μM) against the NCI-H460 cell line. Among them, the IC_50_ value of complex **87** toward the NCI-H460 cell line was 2.8 ± 1.1 μM, which was significantly higher than that of cisplatin (IC_50_, 5.2 ± 2.2 μM). After 48 h of exposure, complex **88** reduced the proliferation rate of NCI-H1573 lung adenocarcinoma cells by 16.46% (Scheme 9) (Pahontu et al., 2016a). Complex **89** is a Pt (II) complex with α-n heterocyclic thiosemicarbazide as ligand, with an IC_50_ value of 79 ± 4 μM for the A549 cell line after 72 h of incubation (Scheme 9) (Matesanz et al., 2018). The IC_50_ values of complex **90** against the A549 cell line were 146.6, 101.7 and 69.28, respectively, after 24, 48 and 72 h of treatment (Scheme 9) (Salehi et al., 2022).

**SCHEME 10:**
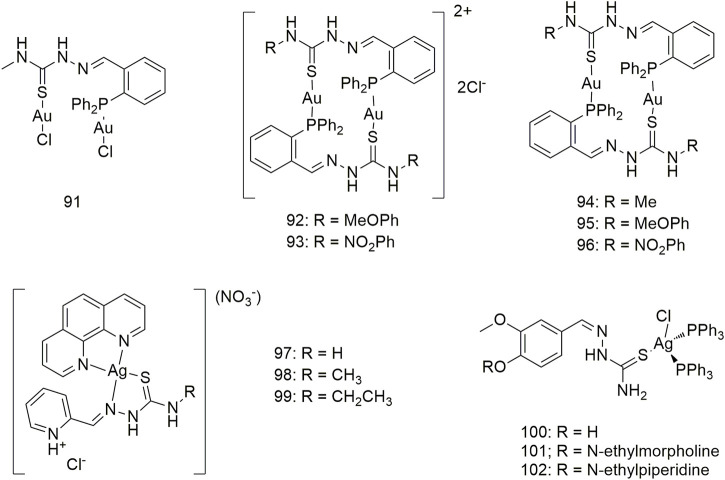
Structures of gold (I) (**90–96**) and silver (I) (**97–102**) complexes of thiosemicarbazone.

Six phosphothiosemicarbazide gold (Au) (I) binuclear complexes (**91–96**, Scheme 10) were prepared using synthetic methods. In terms of *in vitro* cytotoxic activity, the cytotoxic of these Au (I) complexes against the NCI-H460 cell line and MRC5 (normal human lung fibroblasts) was investigated and their IC_50_ values were compared with that of cisplatin (Lessa et al., 2011). Complexes **91** and **94** strongly inhibited thioredoxin reductase activity. Compounds **97–99** not only exhibited good *in vitro* anti-proliferative activity against the A549 cell line with IC_50_ values of 1.49–2.64 μM but also showed less cytotoxicity toward human breast non-tumour cells (MCF-10A) (Silva et al., 2020). The complexes **100–102** showed obvious cytotoxic activity against the A549 cell line, and the IC_50_ values of 48 h incubation were 7.48 ± 0.21, 8.15 ± 1.21 and 6.46 ± 0.51 μM, respectively, which were higher than the cell activity of cisplatin (IC_50_, 23.36 ± 0.42 μM) (Scheme 10).

## Other classes of thiosemicarbazone metal complexes

**SCHEME 11:**
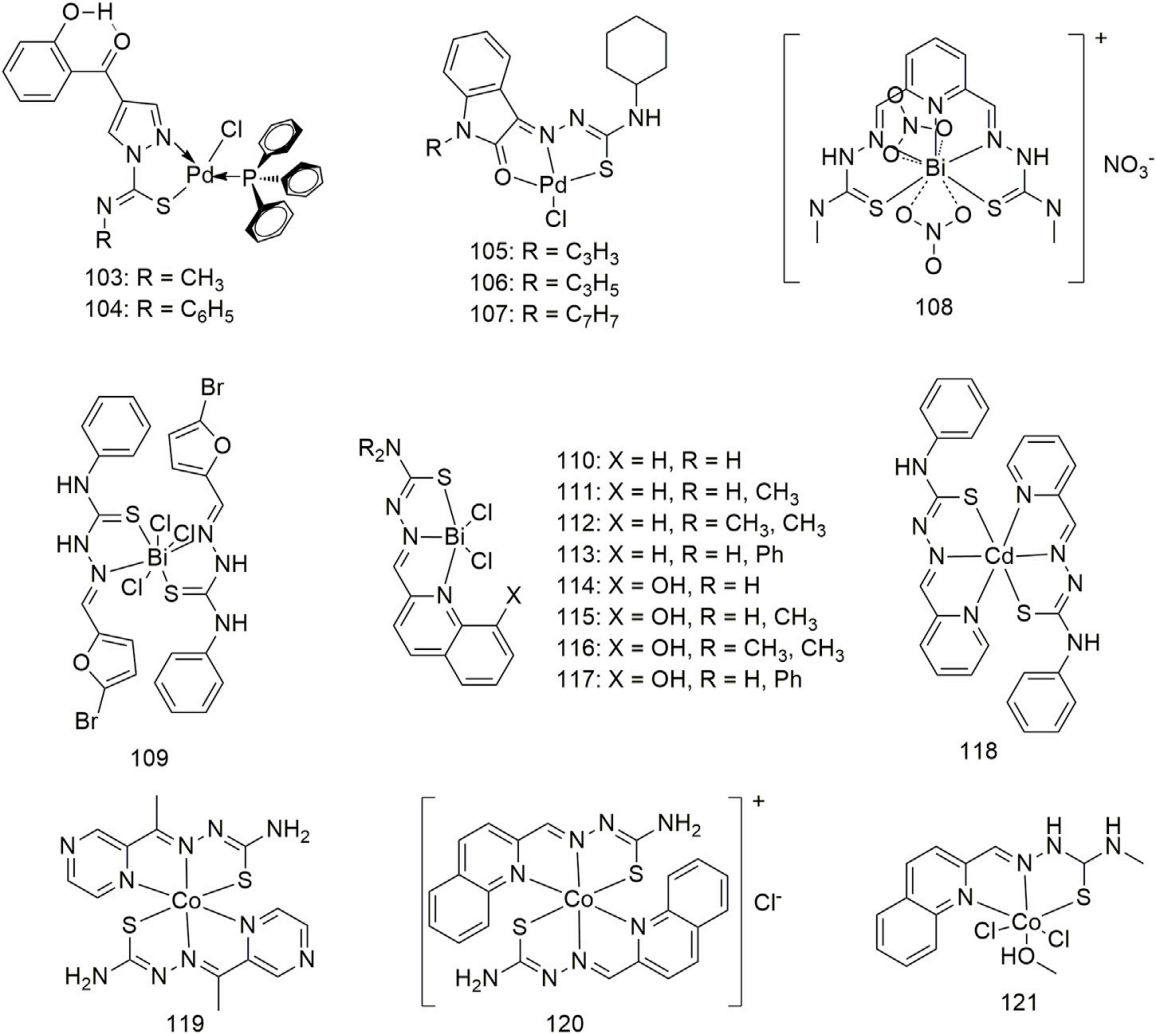
Structures of palladium (II) (**103–107**), bismuth (III) (**108–117**), chromium (III) (**10–18**) and cobalt (II) (**119–121**)complexes of thiosemicarbazone.

Complexes **103** and **104** are two novel pyrazolylthiourea Pd (II) complexes that bind to CT DNA and cleave supercoiled DNA (pUC19) (Scheme 11) (Haribabu et al., 2020). Complexes **103** and **104** show cytotoxic IC_50_ values of 130.3 and 117.2 μM for the A549 cell line, respectively, *in vitro* and are less toxic to normal human lung (IMR90) cells (IC_50_ > 133 μM). Three kinds of clamp-type Pd (II) complexes (**105–107**, Scheme 11) were synthesised by reacting PdCl_2_ with thiourea ligands, which can bind to CT-DNA and BSA. Spectroscopic evidence shows an intercalation pattern between DNA and Pd (II) complexes. The binding mode is further confirmed by CD spectroscopy, suggesting that the binding occurs in a non-grooved mode. Changes in the protein secondary structure by the complex were confirmed by simultaneous fluorescence spectroscopy studies. Spectral evidence indicates that the complex shows good binding properties to proteins. Complex **105–107** cleaved pUC19 plasmid DNA without external reagents. These complexes exhibit significant *in vitro* cytotoxicity against the A549 cell line, suggesting that they can kill cancer cells even at low concentrations. The IC_50_ of complex **107** toward the A549 cell line is 22.92 μM. Complex **108** significantly inhibits the colony formation and migration abilities of the A549 cell line and can induce apoptosis (Scheme 11) (Ouyang et al., 2017). The anti-cancer activity of complex 108 is much higher than that of its parent ligand, and it generates no obvious toxicity toward non-cancerous human lung fibroblasts (HLF). *In vivo* experiments proved that complex **108** shows an obvious inhibitory effect on the growth of the A549-xenografted tumour in tumour-bearing mice and has no adverse effects on the mouse body weight and liver. Complex **109** effectively inhibits the proliferation and migration of the A549 and H460 cell lines and induces cell apoptosis (Scheme 11) (Ruizhuo et al., 2016). Complex **109** has IC_50_ values of 16.41 ± 0.93 μM and 20.04 ± 1.28 μM for the A549 and H460 cell lines, respectively, and demonstrates low cytotoxicity against normal HLF (IC_50_, 117.16 ± 5.96 μM). These results suggest that thiosemicarbazone complexes with bismuth (Bi) (III) may be an interesting and effective anti-lung cancer drug candidate. The coordination of Bi complexes **110–117** is a thiosemicarbazone containing a quinoline group (Scheme 11) (Scaccaglia et al., 2022). Among them, complexes **113** and **114** show significant inhibitory activities against the A549 cell line with IC_50_ values of 5.05 ± 1.79 μM and 46.96 ± 16.66 μM, respectively. Complex **111** exhibits significant inhibitory activity against the A549 cell line (IC_50_, 14.0 ± 1.1 μM) (Abyar et al., 2019). This complex exerts inhibitory activity on the A549 cell line in a cell cycle-dependent manner and induces caspase-mediated apoptosis and caspase-independent cell death. The anti-tumour activity of complex **119** (IC_50_, 18.9 µM) on the A549 cell line was significantly higher than that of the ligand (IC_50_, 265.3 µM) (Ming et al., 2015). Through groove binding, two cobalt (II) complexes (**120** and **121**, Scheme 11) bind to DNA, and the binding capacity of complex **121** is stronger than that of complex **120**. Complex **121** shows higher anti-tumour (A549) activity than complex **120**. Although the inhibitory activity of complex **121** on A549 cells was inferior to that of cisplatin, its inhibitory efficacy on A549/CDDP in drug-resistant cells was higher than that of cisplatin (Xiaorui et al., 2013).

**SCHEME 12:**
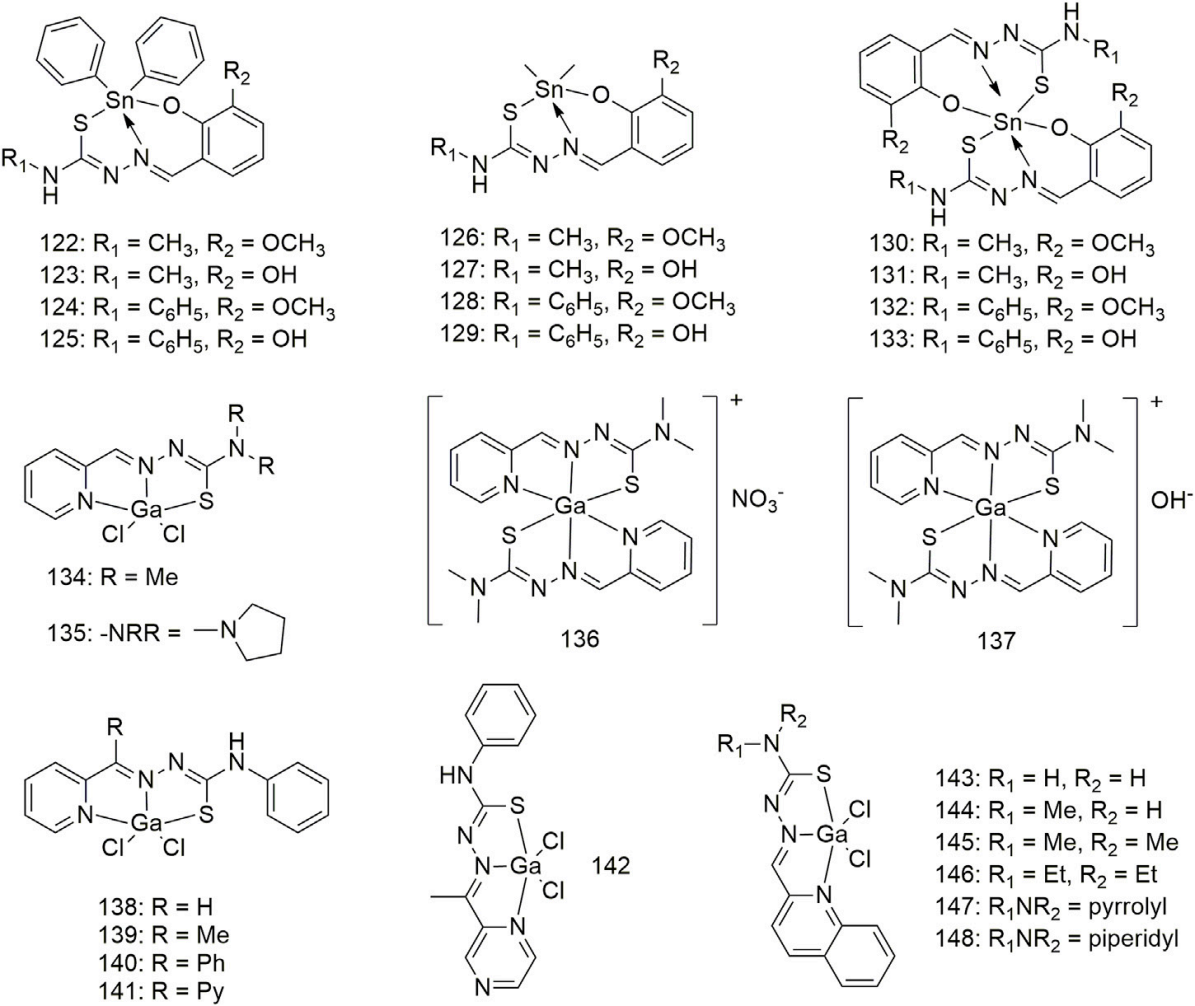
Structures of tin (II) (**122–133**) and gallium (III) (**134–148**) complexes of thiosemicarbazone.

Complexes **122–133** were derived from four thiosemicarbazone Schiff bases (Scheme 12). Except for complex **124**, which has no experimental data, and complex **127**, which is inert, other complexes demonstrate significant anti-lung cancer (H460) activity with IC_50_ values less than 3.2 μM (Yusof et al., 2020). Two types of gallium (Ga) (III) complexes (**134–137**, Scheme 12) with ligand/Ga (III) ratios of 2:1 and 1:1 exhibit significant anti-proliferative activity against the NCI-H460 cell line, and their IC_50_ values are between 0.72 and 0.43 μM. The anti-proliferative activity of Ga (III) complexes with a metal/ligand ratio of 1:1 (complex **135**) was significantly higher than that of the 1:2 (complex **137**) (Qi et al., 2017). Complexes 135 and 137 significantly promote the release of cytochrome C from mitochondria and increase the activities of caspase-3 and -9 in the NCI-H460 cell line. Both types of Ga (III) complexes inhibited the G1/S transition better than ligands alone. Five 1:1 ligand/Ga (III) complexes (**138–142**, Scheme 12) exhibit significant anti-proliferative activity against lung cancer (A549) with IC_50_ values between 0.37 and 2.32 μM. The structure–activity relationship results showed that modifying the lipophilic groups present on the ligands significantly improves its anti-proliferative activity, and this biological activity was further enhanced after coordination with Ga. The anti-cancer (A549) activities (IC_50_, 0.46–1.41 μM) of the Ga (III) complexes **143–148** all exceeded those of the corresponding metal-free ligands (Cao et al., 2019). These Ga (III) complexes are non-toxic to normal hepatocytes and exhibit strong selectivity for tumour cells. The anti-tumour (A549) mechanism of Ga (III) complexes produces intracellular ROS, disrupts mitochondrial membrane potential and ultimately promotes apoptosis.

## Copper, nickel and zinc complexes of the same ligand

**SCHEME 13:**
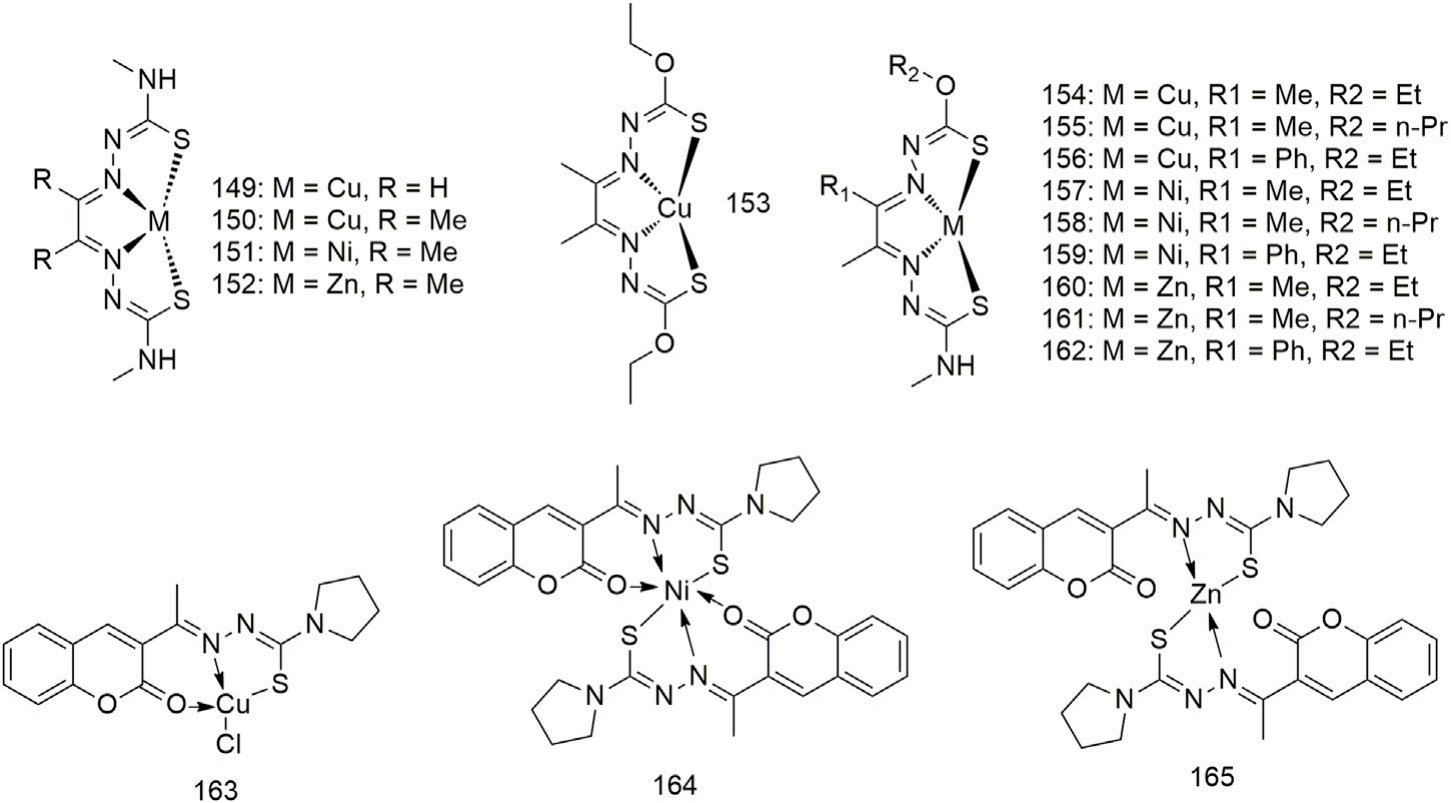
Structures of copper (II), nickel (II) and zinc (II) complexes of thiosemicarbazone.

A series of hybrid ligands derived from 4-methyl-3-thiosemicarbazide and hydrazinecarbothioic acid O-alkyl ester ligands chelate with Cu, Ni and zinc (Zn) to form complexes **149–162** (Scheme 13) (Andres et al., 2020). Cu derivatives **154** and **156** exhibit strong anti-cancer selectivity, having GI_50_ values of less than 100 nM against the A549 lung adenocarcinoma cells, and at least 20-fold lower GI_50_ activity against IMR90 (>2.0 μM) non-malignant lung fibroblasts. While Ni complexes exhibit much lower anti-tumour activity (>5.3 μM) compared with Cu complexes, Zn complexes show weaker activity (0.055–7.6 μM). Zn and Ni complexes demonstrate little cancer selectivity. The complexes Cu (II) (**163**), Ni (II) (**164**) and Zn (II) (**165**) interacts with CT-DNA *via* intercalation binding as well as with BSA (Rahman et al., 2017). The activity of these complexes on the A549 cell line was lower than in the other cell lines, in which complex **165** exhibits moderate cytotoxicity against the A549 cell line with an IC_50_ value of 200 μg/ml.

## Discussion

Recently, the increase in the number of patients with lung cancer and the emergence of tumour resistance, the demand for metal compounds that treat cancer continues to grow, not only because cancer is difficult to cure but also because metal-based compounds with remarkable antitumor activity. Metal-based compounds show high cytotoxicity *in vitro*. Furthermore, the substitution of ligands and modification of existing chemical structures have produced a variety of metal-based compounds, some of which show higher tumour cytotoxicity, low toxicity toward non-tumour cells and better pharmacokinetics.

Metal complexes, such as Cu, Zn, Au, silver (Ag), Pt and Ru, exhibit higher anti-tumour activity than metal-free ligands. Cancer cells have a greater demand for Cu than normal cells, and Cu metabolism is strongly linked to angiogenesis and metastasis (Santini et al., 2014; Shobha et al., 2018; Kannappan et al., 2021). Therefore, since the 1970s, various ligands that form complexes with Cu, such as thiosemicarbazides, imidazoles and phosphines, have been proposed as potential anti-cancer drugs. Furthermore, Cu salts and complexes have been shown to inhibit tumour cell proliferation (Santini et al., 2014). In fact, these Cu complexes allow Cu to undergo redox cycling between the reduced monovalent and oxidised divalent states under the action of cellular oxidants and reductants (Balsa et al., 2021). Cu complexes show redox activity, engender intracellular ROS overload and promote apoptosis through the mitochondrial apoptotic pathway. Many ruthenium (Ru) complexes have been synthesised and evaluated as possible cancer therapeutics over the past two decades (Gatti et al., 2018). The activity of Ru (III) compounds depends on *in vivo* reduction to more reactive Ru (II) substances, which has led to increased interest in organometallic Ru (II) arene complexes in octahedral configurations, where aromatics stabilise Ru in Ru oxidation states (Haribabu et al., 2018). Some semi-sandwiched Ru (II) arene complexes exhibit notable anti-cancer activity *in vitro* and *in vivo* (Gatti et al., 2018). The activity of Ru complex cells is significantly higher than that of cisplatin and the former shows tumour selectivity. This may be due to the good DNA/protein binding affinity of the Ru complex. Ni (II) complexes exhibit obvious inhibitory activity on the A549 cell line and less toxicity on the L929 cell line. Ni (II) complexes interact with CT-DNA and BSA, cleave DNA on the absence of any external factors and change the secondary structure of protein. Nickel can form rich molecular geometries, ensuring the formation of complexes with improved properties (Bisceglie et al., 2019). This facilitates improved drug properties without any increase in cellular resistance or negative side effects from the drugs. Thiosemicarbazone Pt (II), Au (I) and Ag (I) complexes were significantly more active on lung cancer cells than cisplatin. Owing to their similar structural characteristics and coordination chemistry with Pt, palladium (Pd) complexes have been thoroughly investigated for anti-cancer activity similar to that of their Pt analogues. These metal complexes have complicated and diverse action mechanisms and anti-tumour activities, which compensate for the deficiencies in the existing drugs. However, these studies are only at the basic research stage. Overall, a rational design of anti-tumour drugs based on other metal classes is yet to be achieved, and there is a lack of unique metal complexes. Although thiosemicarbazone-based metal complexes have been used in the laboratory and have exhibited good outcomes on lung cancer cells and tumour-bearing nude mice, achieving a clear distinction between therapeutic and toxic doses is a major challenge.

## References

[B1] Abyar S. Khandar A. A. Salehi R. Abolfazl H. S. Alizadeh E. Mahkam M. (2019). *In vitro* nephrotoxicity and anticancer potency of newly synthesized cadmium complexes. Sci. Rep. 9 (1), 14686. 10.1038/s41598-019-51109-9 31604983 PMC6789105

[B2] Andres S. A. Bajaj K. Vishnosky N. S. Peterson M. A. Mashuta M. S. Buchanan R. M. (2020). Synthesis, characterization, and biological activity of hybrid thiosemicarbazone-alkylthiocarbamate metal complexes. Inorg. Chem. 59 (7), 4924–4935. 10.1021/acs.inorgchem.0c00182 32159342

[B3] Aneesrahman K. N. Ramaiah K. Rohini G. Stefy G. P. Bhuvanesh N. S. P. Sreekanth A. (2019). Synthesis and characterisations of copper (II) complexes of 5-methoxyisatin thiosemicarbazones: Effect of N-terminal substitution on DNA/protein binding and biological activities. Inorganica Chim. Acta 492, 131–141. 10.1016/j.ica.2019.04.019

[B4] Balsa L. M. Baran E. J. Leon I. E. (2021). Copper complexes as antitumor agents: *In vitro* and *in vivo* evidences. Curr. Med. Chem. 28. E-pub ahead of print. 10.2174/0929867328666211117094550 34789122

[B5] Barrera-Rodriguez R. Fuentes J. M. (2015). Multidrug resistance characterization in multicellular tumour spheroids from two human lung cancer cell lines. Cancer Cell Int. 15, 47. 10.1186/s12935-015-0200-6 26221079 PMC4517505

[B6] Bisceglie F. Orsoni N. Pioli M. Bonati B. Tarasconi P. Rivetti C. (2019). Cytotoxic activity of copper(ii), nickel(ii) and platinum(ii) thiosemicarbazone derivatives: Interaction with DNA and the H2A histone peptide. Metallomics 11 (10), 1729–1742. 10.1039/c9mt00166b 31502621

[B7] Brody H. (2014). Lung cancer. Nature 513 (7517), S1. 10.1038/513S1a 25208065

[B8] Cao W. Qi J. Qian K. Tian L. Cheng Z. Wang Y. (2019). Structure-activity relationships of 2quinolinecarboxaldehyde thiosemicarbazone gallium(III) complexes with potent and selective anticancer activity. J. Inorg. Biochem. 191, 174–182. 10.1016/j.jinorgbio.2018.11.017 30530178

[B10] de Siqueira L. de Moraes G. P. de Lima F. L. de Melo R. M. Leite A. (2019a). Multi-target compounds acting in cancer progression: Focus on thiosemicarbazone, thiazole and thiazolidinone analogues. Eur. J. Med. Chem. 170, 237–260. 10.1016/j.ejmech.2019.03.024 30904782

[B11] Dharmasivam M. Azad M. G. Afroz R. Richardson V. Jansson P. J. Richardson D. R. (2022). The thiosemicarbazone, DpC, broadly synergizes with multiple anti-cancer therapeutics and demonstrates temperature- and energy-dependent uptake by tumor cells. Biochim. Biophys. Acta. Gen. Subj. 1866 (8), 130152. 10.1016/j.bbagen.2022.130152 35436509

[B12] Gatti A. Habtemariam A. Romero-Canelon I. Song J. I. Heer B. Clarkson G. J. (2018). Half-sandwich arene ruthenium(II) and osmium(II) thiosemicarbazone complexes: Solution behavior and antiproliferative activity. Organometallics 37 (6), 891–899. 10.1021/acs.organomet.7b00875 29681675 PMC5908187

[B13] Gu S. Yu P. Hu J. Liu Y. Li Z. Qian Y. (2019). Mitochondria-localizing N-heterocyclic thiosemicarbazone copper complexes with good cytotoxicity and high antimetastatic activity. Eur. J. Med. Chem. 164, 654–664. 10.1016/j.ejmech.2019.01.014 30641446

[B14] Haribabu J. Alajrawy O. I. Jeyalakshmi K. Balachandran C. Krishnan D. A. Bhuvanesh N. (2021). N-substitution in isatin thiosemicarbazones decides nuclearity of Cu(II) complexes - spectroscopic, molecular docking and cytotoxic studies. Spectrochim. Acta. A Mol. Biomol. Spectrosc. 246, 118963. 10.1016/j.saa.2020.118963 33017789

[B15] Haribabu J. Balachandran C. Tamizh M. M. Arun Y. Bhuvanesh N. Aoki S. (2020). Unprecedented formation of palladium(II)-pyrazole based thiourea from chromone thiosemicarbazone and [PdCl2(PPh3)2]: Interaction with biomolecules and apoptosis through mitochondrial signaling pathway. J. Inorg. Biochem. 205, 110988. 10.1016/j.jinorgbio.2019.110988 31981770

[B17] Haribabu J. Jeyalakshmi K. Arun Y. Bhuvanesh N. Perumal P. T. Karvembu R. (2017). Synthesis of Ni(II) complexes bearing indole-based thiosemicarbazone ligands for interaction with biomolecules and some biological applications. J. Biol. Inorg. Chem. 22 (4), 461–480. 10.1007/s00775-016-1424-1 27995332

[B18] Haribabu J. Jeyalakshmi K. Arun Y. Bhuvanesh N. S. P. Perumal P. T. Karvembu R. (2015). Synthesis, DNA/protein binding, molecular docking, DNA cleavage and *in vitro* anticancer activity of nickel(II) bis(thiosemicarbazone) complexes. RSC Adv. 5 (57), 46031–46049. 10.1039/C5RA04498G

[B19] Haribabu J. Sabapathi G. Tamizh M. M. Balachandran C. Bhuvanesh N. S. P. Venuvanalingam P. (2018). Water-soluble Mono- and binuclear Ru(η6-p-cymene) complexes containing indole thiosemicarbazones: Synthesis, DFT modeling, biomolecular interactions, and *in vitro* anticancer activity through apoptosis. Organometallics 37 (8), 1242–1257. 10.1021/acs.organomet.8b00004

[B20] Kalaiarasi G. Dharani S. Rajkumar S. Lynch V. M. Prabhakaran R. (2020). Binuclear Ni(II) complexes containing ONS donor Schiff base ligands: Preparation, spectral characterization, X-ray crystallography and biological exploration. J. Inorg. Biochem. 211, 111176. 10.1016/j.jinorgbio.2020.111176 32730983

[B21] Kalaiarasi G. Rajkumar S. R. J. Dharani S. Fronczek F. R. Muthukumar N. M. S. A. Prabhakaran R. (2018). Cyclometallated ruthenium(ii) complexes with 3-acetyl-2[H]-chromene-2-one derived CNS chelating ligand systems: Synthesis, X-ray characterization and biological evaluation. New J. Chem. 42 (1), 336–354. 10.1039/C7NJ02877F

[B22] Kalaivani P. Prabhakaran R. Dallemer F. Vaishnavi E. Poornima P. Padma V. V. (2014a). Synthesis, structural characterization, DNA/Protein binding and *in vitro* cytotoxicity of isomeric ruthenium carbonyl complexes. J. Organomet. Chem. 762, 67–80. 10.1016/j.jorganchem.2014.04.003

[B23] Kalaivani P. Saranya S. Poornima P. Prabhakaran R. Dallemer F. Vijaya P. V. (2014). Biological evaluation of new nickel(II) metallates: Synthesis, DNA/protein binding and mitochondrial mediated apoptosis in human lung cancer cells (A549) via ROS hypergeneration and depletion of cellular antioxidant pool. Eur. J. Med. Chem. 82, 584–599. 10.1016/j.ejmech.2014.05.075 24946146

[B24] Kannappan V. Ali M. Small B. Rajendran G. Elzhenni S. Taj H. (2021). Recent advances in repurposing disulfiram and disulfiram derivatives as copper-dependent anticancer agents. Front. Mol. Biosci. 8, 741316. 10.3389/fmolb.2021.741316 34604310 PMC8484884

[B25] Kowol C. R. Trondl R. Heffeter P. Arion V. B. Jakupec M. A. Roller A. (2009). Impact of metal coordination on cytotoxicity of 3-aminopyridine-2-carboxaldehyde thiosemicarbazone (triapine) and novel insights into terminal dimethylation. J. Med. Chem. 52 (16), 5032–5043. 10.1021/jm900528d 19637923

[B26] Lessa J. A. Guerra J. C. de Miranda L. F. Romeiro C. F. Da S. J. Mendes I. C. (2011). Gold(I) complexes with thiosemicarbazones: Cytotoxicity against human tumor cell lines and inhibition of thioredoxin reductase activity. J. Inorg. Biochem. 105 (12), 1729–1739. 10.1016/j.jinorgbio.2011.09.008 22005449

[B27] Lin X. D. Liu Y. H. Xie C. Z. Bao W. G. Shen J. Xu J. Y. (2017). Three Pt(ii) complexes based on thiosemicarbazone: Synthesis, HSA interaction, cytotoxicity, apoptosis and cell cycle arrest. RSC Adv. 7 (42), 26478–26486. 10.1039/C7RA04443G

[B28] Liu Y. H. Li A. Shao J. Xie C. Z. Song X. Q. Bao W. G. (2016). Four Cu(ii) complexes based on antitumor chelators: Synthesis, structure, DNA binding/damage, HSA interaction and enhanced cytotoxicity. Dalton Trans. 45 (19), 8036–8049. 10.1039/c6dt00451b 27071545

[B30] Lovejoy D. B. Sharp D. M. Seebacher N. Obeidy P. Prichard T. Stefani C. (2012). Novel second-generation di-2-pyridylketone thiosemicarbazones show synergism with standard chemotherapeutics and demonstrate potent activity against lung cancer xenografts after oral and intravenous administration *in vivo* . J. Med. Chem. 55 (16), 7230–7244. 10.1021/jm300768u 22861499

[B31] Mahale S. Bharate S. B. Manda S. Joshi P. Jenkins P. R. Vishwakarma R. A. (2015). Antitumour potential of BPT: A dual inhibitor of cdk4 and tubulin polymerization. Cell Death Dis. 6, e1743. 10.1038/cddis.2015.96 25950473 PMC4669722

[B32] Malarz K. Mrozek-Wilczkiewicz A. Serda M. Rejmund M. Polanski J. Musiol R. (2018). The role of oxidative stress in activity of anticancer thiosemicarbazones. Oncotarget 9 (25), 17689–17710. 10.18632/oncotarget.24844 29707141 PMC5915149

[B33] Maqbool S. N. Lim S. C. Park K. C. Hanif R. Richardson D. R. Jansson P. J. (2020). Overcoming tamoxifen resistance in oestrogen receptor-positive breast cancer using the novel thiosemicarbazone anti-cancer agent, DpC. Br. J. Pharmacol. 177 (10), 2365–2380. 10.1111/bph.14985 31975484 PMC7174880

[B34] Matesanz A. I. Herrero J. M. Quiroga A. G. (2021a). Chemical and biological evaluation of thiosemicarbazone-bearing heterocyclic metal complexes. Curr. Top. Med. Chem. 21 (1), 59–72. 10.2174/1568026620666201022144004 33092510

[B35] Matesanz A. I. Jimenez F. E. Ruiz M. C. Balsa L. M. Navarro R. C. León I. E. (2018). Mononuclear Pd(ii) and Pt(ii) complexes with an α-N-heterocyclic thiosemicarbazone: Cytotoxicity, solution behaviour and interaction versus proven models from biological media. Inorg. Chem. Front. 5 (1), 73–83. 10.1039/C7QI00446J

[B36] Mathiyan M. Jebiti H. Nattamai S. P. B. Ramasamy K. Anandaram S. (2016). Synthesis, X-ray crystal structure, DNA/protein binding, DNA cleavage and cytotoxicity studies of N(4) substituted thiosemicarbazone based copper(II)/nickel(II) complexes. Inorg. Chim. Acta. 449, 82–95. 10.1016/j.ica.2016.04.043

[B38] Mckenzie-Nickson S. Bush A. I. Barnham K. J. (2016). Bis(thiosemicarbazone) metal complexes as therapeutics for neurodegenerative diseases. Curr. Top. Med. Chem. 16 (27), 3058–3068. 10.2174/1568026616666160216155746 26881708

[B39] Ming X. L. Jing Z. Zi L. W. Jing P. W. (2015). Synthesis, crystal structure and antitumor study of a cobalt(II) complex of the 2-acetylpyrazine thiosemicarbazone. Z. für Naturforsch. B 63 (1), 1–5. 10.1515/znb-2008-0101

[B40] Muralisankar M. Dheepika R. Haribabu J. Balachandran C. Aoki S. Bhuvanesh N. (2019). Design, synthesis, DNA/HSA binding, and cytotoxic activity of half-sandwich Ru(II)-Arene complexes containing triarylamine-thiosemicarbazone hybrids. ACS Omega 4 (7), 11712–11723. 10.1021/acsomega.9b01022 31460277 PMC6682138

[B41] Onodera K. Kasuga N. C. Takashima T. Hara A. Amano A. Murakami H. (2007). Synthesis, reaction and structure of a highly light-stable silver(I) cluster with an Ag4S4N4 core having a tridentate 4N-morpholyl 2-acetylpyridine thiosemicarbazone ligand: Use of water-soluble silver(I) carboxylates as a silver(I) source. Dalton Trans. 33, 3646–3652. 10.1039/b702427d 17700827

[B42] Ouyang R. Yang Y. Tong X. Feng K. Yang Y. Tao H. (2017). Potent anticancer activity of a new bismuth (III) complex against human lung cancer cells. J. Inorg. Biochem. 168, 18–26. 10.1016/j.jinorgbio.2016.12.006 28006662

[B43] Pahontu E. Paraschivescu C. Ilies D. C. Poirier D. Oprean C. Paunescu V. (2016a). Synthesis and characterization of novel Cu(II), Pd(II) and Pt(II) complexes with 8-Ethyl-2-hydroxytricyclo(7.3.1.0(2, 7))tridecan-13-one-thiosemicarbazone: Antimicrobial and *in vitro* antiproliferative activity. Molecules 21 (5), E674. 10.3390/molecules21050674 27213326 PMC6273217

[B45] Qi J. Deng J. Qian K. Tian L. Li J. He K. (2017). Novel 2-pyridinecarboxaldehyde thiosemicarbazones Ga(III) complexes with a high antiproliferative activity by promoting apoptosis and inhibiting cell cycle. Eur. J. Med. Chem. 134, 34–42. 10.1016/j.ejmech.2017.04.009 28395152

[B46] Qi J. Liang S. Gou Y. Zhang Z. Zhou Z. Yang F. (2015). Synthesis of four binuclear copper(II) complexes: Structure, anticancer properties and anticancer mechanism. Eur. J. Med. Chem. 96, 360–368. 10.1016/j.ejmech.2015.04.031 25899339

[B47] Qi J. Wang X. Liu T. Kandawa S. M. Wang Y. Zheng X. (2020). Synthesis, antiproliferative activity and mechanism of copper(II)-thiosemicarbazone complexes as potential anticancer and antimicrobial agents. J. Coord. Chem. 73 (7), 1208–1221. 10.1080/00958972.2020.1768378

[B49] Qi J. Yao Q. Tian L. Wang Y. (2018). Piperidylthiosemicarbazones Cu(II) complexes with a high anticancer activity by catalyzing hydrogen peroxide to degrade DNA and promote apoptosis. Eur. J. Med. Chem. 158, 853–862. 10.1016/j.ejmech.2018.09.034 30248656

[B50] Qi J. Zhang Y. Gou Y. Zhang Z. Zhou Z. Wu X. (2016). Developing an anticancer copper(II) pro-drug based on the His242 residue of the human serum albumin carrier IIA subdomain. Mol. Pharm. 13 (5), 1501–1507. 10.1021/acs.molpharmaceut.5b00938 27017838

[B51] Rahman K. A. Haribabu J. Balachandran C. Bhuvanesh N. S. Karvembu R. Sreekanth A. (2017). Copper, nickel and zinc complexes of 3-acetyl coumarin thiosemicarbazone: Synthesis, characterization and *in vitro* evaluation of cytotoxicity and DNA/protein binding properties. Polyhedron 135, 26–35. 10.1016/j.poly.2017.06.044

[B52] Rao V. A. Klein S. R. Agama K. K. Toyoda E. Adachi N. Pommier Y. (2009). The iron chelator Dp44mT causes DNA damage and selective inhibition of topoisomerase IIalpha in breast cancer cells. Cancer Res. 69 (3), 948–957. 10.1158/0008-5472.CAN-08-1437 19176392 PMC7322628

[B53] Rostan S. Mahler G. Otero L. (2021). Selenosemicarbazone metal complexes as potential metal-based drugs. Curr. Med. Chem. 29. E-pub ahead of print. 10.2174/0929867329666211222115035 34951353

[B54] Ruizhuo O. Yang Y. Xiao T. Yaoqin Y. Huihong T. Tianyu Z. (2016). Potential anti-cancer activity of a novel Bi(III) containing thiosemicarbazone derivative. Inorg. Chem. Commun. 73, 138–141. 10.1016/j.inoche.2016.10.020

[B55] Salehi R. Abyar S. Ramazani F. Khandar A. A. Hosseini-Yazdi S. A. White J. M. (2022). Enhanced anticancer potency with reduced nephrotoxicity of newly synthesized platin-based complexes compared with cisplatin. Sci. Rep. 12 (1), 8316. 10.1038/s41598-022-11904-3 35585092 PMC9117324

[B56] Santini C. Pellei M. Gandin V. Porchia M. Tisato F. Marzano C. (2014). Advances in copper complexes as anticancer agents. Chem. Rev. 114 (1), 815–862. 10.1021/cr400135x 24102434

[B57] Scaccaglia M. Rega M. Bacci C. Giovanardi D. Pinelli S. Pelosi G. (2022). Bismuth complex of quinoline thiosemicarbazone restores carbapenem sensitivity in NDM-1-positive *Klebsiella pneumoniae* . J. Inorg. Biochem. 234, 111887. 10.1016/j.jinorgbio.2022.111887 35690039

[B58] Scarim C. B. Chin C. M. (2022a). Recent trends in drug development for the treatment of adenocarcinoma breast cancer: Thiazole, triazole, and thiosemicarbazone analogues as efficient scaffolds. Anticancer. Agents Med. Chem. 22 (12), 2204–2240. 10.2174/1871520621666211201152815 34852749

[B60] Serda M. Kalinowski D. S. Rasko N. Potuckova E. Mrozek-Wilczkiewicz A. Musiol R. (2014). Exploring the anti-cancer activity of novel thiosemicarbazones generated through the combination of retro-fragments: Dissection of critical structure-activity relationships. PLoS One 9 (10), e110291. 10.1371/journal.pone.0110291 25329549 PMC4199632

[B61] Shobha D. C. Thulasiram B. Aerva R. R. Nagababu P. (2018). Recent advances in copper intercalators as anticancer agents. J. Fluoresc. 28 (5), 1195–1205. 10.1007/s10895-018-2283-7 30171479

[B62] Silva D. Becceneri A. B. Santiago J. Gomes N. J. Ellena J. Cominetti M. R. (2020). Silver(I) complexes of 3-methoxy-4-hydroxybenzaldehyde thiosemicarbazones and triphenylphosphine: Structural, cytotoxicity, and apoptotic studies. Dalton Trans. 49 (45), 16474–16487. 10.1039/d0dt01134g 32914824

[B63] Stariat J. Kovarikova P. Kucera R. Klimes J. Kalinowski D. S. Richardson D. R. (2013). Identification of *in vitro* metabolites of the novel anti-tumor thiosemicarbazone, DpC, using ultra-high performance liquid chromatography-quadrupole-time-of-flight mass spectrometry. Anal. Bioanal. Chem. 405 (5), 1651–1661. 10.1007/s00216-012-6562-x 23180090

[B64] Stefani C. Jansson P. J. Gutierrez E. Bernhardt P. V. Richardson D. R. Kalinowski D. S. (2013). Alkyl substituted 2'-benzoylpyridine thiosemicarbazone chelators with potent and selective anti-neoplastic activity: Novel ligands that limit methemoglobin formation. J. Med. Chem. 56 (1), 357–370. 10.1021/jm301691s 23276209

[B65] Summers K. L. (2019a). A structural chemistry perspective on the antimalarial properties of thiosemicarbazone metal complexes. Mini Rev. Med. Chem. 19 (7), 569–590. 10.2174/1389557518666181015152657 30324878

[B66] Sung H. Ferlay J. Siegel R. L. Laversanne M. Soerjomataram I. Jemal A. (2021). Global cancer statistics 2020: GLOBOCAN estimates of incidence and mortality worldwide for 36 cancers in 185 countries. Ca. Cancer J. Clin. 71 (3), 209–249. 10.3322/caac.21660 33538338

[B67] Tsai H. I. Jiang L. Zeng X. Chen H. Li Z. Cheng W. (2018). DACHPt-loaded nanoparticles self-assembled from biodegradable dendritic copolymer polyglutamic acid-b-D-alpha-tocopheryl polyethylene glycol 1000 succinate for multidrug resistant lung cancer therapy. Front. Pharmacol. 9, 119. 10.3389/fphar.2018.00119 29515445 PMC5826327

[B68] Wang J. Gou Y. Zhang Z. Yu P. Qi J. Qin Q. (2018). Developing an anticancer copper(II) multitarget pro-drug based on the His146 residue in the IB subdomain of modified human serum albumin. Mol. Pharm. 15 (6), 2180–2193. 10.1021/acs.molpharmaceut.8b00045 29722993

[B69] Wangpu X. Lu J. Xi R. Yue F. Sahni S. Park K. C. (2016). Targeting the metastasis suppressor, N-myc downstream regulated gene-1, with novel di-2-pyridylketone thiosemicarbazones: Suppression of tumor cell migration and cell-collagen adhesion by inhibiting focal adhesion kinase/paxillin signaling. Mol. Pharmacol. 89 (5), 521–540. 10.1124/mol.115.103044 26895766 PMC4851300

[B70] Wu S. Guo W. Teraishi F. Pang J. Kaluarachchi K. Zhang L. (2006). Anticancer activity of 5-benzylidene-2-phenylimino-1, 3-thiazolidin-4-one (BPT) analogs. Med. Chem. 2 (6), 597–605. 10.2174/1573406410602060597 17105441

[B71] Xiaorui F. Juanjuan D. Rui M. Yun C. Xiaoyi Y. Jianliang Z. (2013). Cobalt(II) complexes with thiosemicarbazone as potential antitumor agents: Synthesis, crystal structures, DNA interactions, and cytotoxicity. J. Coord. Chem. 66 (24), 4268–4279. 10.1080/00958972.2013.867030

[B72] Yu Y. Kalinowski D. S. Kovacevic Z. Siafakas A. R. Jansson P. J. Stefani C. (2009). Thiosemicarbazones from the old to new: Iron chelators that are more than just ribonucleotide reductase inhibitors. J. Med. Chem. 52 (17), 5271–5294. 10.1021/jm900552r 19601577

[B73] Yu Y. Suryo R. Y. Hawkins C. L. Richardson D. R. (2011). The potent and novel thiosemicarbazone chelators di-2-pyridylketone-4, 4-dimethyl-3-thiosemicarbazone and 2-benzoylpyridine-4, 4-dimethyl-3-thiosemicarbazone affect crucial thiol systems required for ribonucleotide reductase activity. Mol. Pharmacol. 79 (6), 921–931. 10.1124/mol.111.071324 21389104

[B74] Yu Y. Wong J. Lovejoy D. B. Kalinowski D. S. Richardson D. R. (2006). Chelators at the cancer coalface: Desferrioxamine to triapine and beyond. Clin. Cancer Res. 12 (23), 6876–6883. 10.1158/1078-0432.CCR-06-1954 17145804

[B75] Yusof E. N. M. Page A. J. Sakoff J. A. Simone M. I. Veerakumarasivam A. Tiekink E. R. (2020). Tin(IV) compounds of tridentate thiosemicarbazone Schiff bases: Synthesis, characterization, *in-silico* analysis and *in vitro* cytotoxicity. Polyhedron 189, 114729. 10.1016/j.poly.2020.114729

[B76] Zhao Y. Guo C. Wang L. Wang S. Li X. Jiang B. (2017). A novel fluorinated thiosemicarbazone derivative- 2-(3, 4-difluorobenzylidene) hydrazinecarbothioamide induces apoptosis in human A549 lung cancer cells via ROS-mediated mitochondria-dependent pathway. Biochem. Biophys. Res. Commun. 491 (1), 65–71. 10.1016/j.bbrc.2017.07.042 28698138

